# Impact of Oropharyngeal Administration of Colostrum in Preterm Newborns’ Oral Microbiome

**DOI:** 10.3390/nu13124224

**Published:** 2021-11-24

**Authors:** Ramon V. Cortez, Andrea Fernandes, Luiz Gustavo Sparvoli, Marina Padilha, Rubens Feferbaum, Corintio Mariani Neto, Carla R. Taddei

**Affiliations:** 1 Department of Clinical Analysis and Toxicology, School of Pharmaceutical Sciences, University of São Paulo, São Paulo 05508-000, Brazil; ramon.cortez@usp.br (R.V.C.); luiz.sparvoli@usp.br (L.G.S.); 2 Human Milk Bank, Hospital Maternidade Leonor Mendes de Barros, São Paulo 03015-000, Brazil; andreapsfernandes@hotmail.com (A.F.); mariani.ops@terra.com.br (C.M.N.); 3 Department of Social and Applied Nutrition, Federal University of Rio de Janeiro, Rio de Janeiro 21941-590, Brazil; marypadilha@gmail.com; 4 Children’s Institute, University of São Paulo, Rua Tremembé, São Paulo 01256-010, Brazil; rfeferbaum@uol.com.br; 5 School of Arts, Sciences and Humanity, University of São Paulo, São Paulo 03828-000, Brazil

**Keywords:** premature newborns, oral microbiota, colostrum, infant nutrition, 16S rRNA

## Abstract

The initial colonization of the human microbiota is of paramount importance. In this context, the oropharyngeal administration of colostrum is a safe, viable, and well-tolerated practice even by the smallest preterm infants. Therefore, this study evaluated the effects of oropharyngeal administration of colostrum on the establishment of preterm infants’ oral microbiota. A longitudinal observational study was carried out with 20 premature neonates, divided into two groups: one receiving the protocol (Oropharyngeal Administration of Colostrum; OAC) and the other one receiving Standard Caare (SC). Saliva samples were collected from the newborns weekly during the study period (from the day of birth until the 21st day of life) for analysis of oral microbiota through 16S rRNA gene sequencing. We observed that the colonization of the oral microbiota of preterm newborns preseanted a higher relative abundance of *Staphylococcus* on the 7th day of life, mainly in the OAC group. Additionally, an increased abundance of *Bifidobacterium* and *Bacteroides* was observed in the OAC group at the first week of life. Regarding alpha and beta diversity, time was a key factor in the oral modulation of both groups, showing how dynamic this environment is in early life.

## 1. Introduction

Colonization of the human microbiota starts in intrauterine life, through amniotic fluid, uterine membranes, and meconium [1,2], contributing to the development of the neonatal oral microbiota that will colonize the rest of the gastrointestinal tract (GIT) [3]. The composition of the oral microbiota in neonates is an important factor for the development of immunity and health, as changes in this community can predispose individuals to the onset of infections or diseases in early life [4,5]. Unlike the oral microbiota of adults, which is stable, the newborn’s oral microbiota is more dynamic and influenced by the food offered in the first months of life [6].

Particularly for premature newborns, breast milk is responsible for transferring beneficial bacterial genera, in addition to oligosaccharides, immunoglobulins, proteins such as lysozyme and lactoferrin, growth factors and cytokines [7,8,9,10,11], contributing to the immune system development.

The composition of breast milk is different in mothers of preterm and full-term newborns [12,13], adapting to the needs of the newborn. The colostrum of mothers who had preterm delivery presents higher quantities of protein and a similar pattern of human milk oligosaccharides (HMOs), comparing to the colostrum of mothers who had term delivery [14].

For premature and/or low-birth-weight newborns, it is not always possible to feed with the own mother’s milk. In this case, the World Health Organization currently recommends the use of pasteurized donor milk for low-birth-weight newborns when the mother’s milk is not available [15]. Although pasteurization promotes the inactivation of some cellular components present in breast milk, such as interferon gamma (IFNγ), tumor necrosis factor alpha (TNF-α), interleukin 1 beta (IL-1β), interleukin 10 (IL-10), and hepatocyte growth factor (HGF) [16], the concentration of HMOs is unchanged [17]. The presence of HMOs, a group of more than 200 types of non-digestible sugars, is known to be extremely important for the development of newborns [18].

Premature neonates remain hospitalized for some time in intensive care units (ICUs), and the administration of a trophic enteral diet is often limited because of the immaturity of the digestive system or the clinical status of the newborn. Given the immunological benefits of providing maternal colostrum, alternative administration techniques have been adopted, including the oropharyngeal administration of colostrum, an immune therapy in which a small amount of colostrum is placed in the oral mucosa for absorption through a sterile cotton swab or oral applicator [19].

The oropharyngeal administration of colostrum is a safe, viable, and well-tolerated practice even by extremely preterm infants [20] and preliminary evidence supports the effect of this practice in reducing time to full enteral feeding [19]. Studies have already observed the immunological effects of this protocol in extremely preterm infants, finding an increase in secretory immunoglobulin A (IgA) and a lower incidence of clinical sepsis [21]. Additionally, they have also observed that the levels of IgA, immunoglobulin M (IgM), resistin, and lactoferrin increased in preterm infants receiving oral administration of colostrum after 30 days [22]. Despite this, few studies have observed the development of oral microbiota in preterm infants undergoing this technique. Thus, the present study evaluated the first weeks of development of the oral microbiota of preterm newborns receiving oropharyngeal colostrum, compared to a control group receiving standard care.

## 2. Materials and Methods

### 2.1. Subjects and Study Design

This longitudinal and observational study was conducted between March and June 2019 and enrolled 24 premature newborns from the Hospital and Maternity Leonor Mendes de Barros (Sao Paulo, Brazil). The present study was approved by the Ethics Committee of the Hospital Maternidade Leonor Mendes de Barros (CAAE: 76151717.0.3001.0063) and by the Faculty of Pharmaceutical Sciences of the University of Sao Paulo (CAAE: 76151717.0.0000.0067).

Newborns were selected when admitted to the neonatal intensive care unit (NICU) to determine eligibility. Premature neonates, with gestational age between 28 and 35 weeks of gestation at birth, who did not present malformations or genetic syndromes, as well as any other type of anomaly, were included in this study. Neonates who died during hospitalization were excluded from the study.

After receiving detailed written information about the nature, objectives, procedures, risks, benefits, and relevance of the study, the infants’ parents signed an Informed Consent Form (ICF). The procedures were carried out in accordance with the Declaration of Helsinki and the ethical standards of the Brazilian Ministry of Health (National Health Council—Resolution No. 466/2012).

### 2.2. Group Definition and Oropharyngeal Administration of Colostrum Protocol

The oropharyngeal administration of colostrum is an oral immunotherapy aimed at premature newborns admitted to the Neonatal Intensive Care Unit (NICU), selected by the medical team of the unit. The research team did not participate in the choice, prescription, and administration of either the colostrum protocol or the diet of the newborns participating in the study. In this study, the practice started between the first 24 to 48 h of life and continued for at least 72 consecutive hours (3 days). In total, 0.2 mL of colostrum was administered in a 1-mL syringe, which was instilled in the newborn’s oral cavity every 3 h. During maternal hospitalization, colostrum was collected in the human milk bank (HMB). If the mother was unable to get out of bed, the HMB team carried out the colostrum collection in the maternal hospitalization unit and then took the colostrum to the NICU in a cold chain. If the mother’s milk was not available, pasteurized milk from a donor from the HMB was administered. Holder pasteurization method was used, as recommended by the Brazilian Network of Human Milk Banks [23]. 

All newborns included in this study received standard care provided by the hospital’s medical staff, according to their stability and degree of prematurity. In addition to standard care, oropharyngeal administration of colostrum has been prescribed for some newborns. Thus, the standard care group (SC) was composed of newborns who did not receive colostrum administration and the group that received the protocol was called oropharyngeal administration of colostrum (OAC).

### 2.3. Sample Collection and DNA Isolation

The first saliva sample was collected within the first 24 h after birth (T0) and subsequent samples were collected weekly, on the 7th (T1), 14th (T2), and 21st (T3) days after birth, using two sterile cotton swabs, carefully rubbing the newborn’s cheeks and tongue for approximately 30 s. The swab was then immediately transferred to a tube containing 500 µL of Phosphate Buffered Saline (PBS) (pH 7.4) and stored at −20 °C until the sample was sent to the Molecular Biology Laboratory, what occurred within 24 h after collection. In the laboratory, the samples were stored at −80 °C until the time of DNA extraction.

The total DNA of the saliva samples was extracted according to the QIAamp DNA Blood Mini Kit protocol (QIAGEN, Hilden, Germany), following the manufacturer’s instructions, with minor adaptations. First, the tube containing the swab and 500 µL of PBS (pH 7.4) was shaken at maximum speed for 30 s. Then, 500 µL of 0.1% dithiothreitol (DTT, Invitrogen), a reducing agent, was added to decrease the sample viscosity, vortexed for 1 min and then incubated for 10 min at room temperature. The suspension was then centrifuged at 15,700× *g* for 15 min. The supernatant was discarded, and the pellet was lysed in 200 µL of TELS buffer (20mg/mL lysozyme: 1M Tris-HCl (pH 7.5), 0.5 M EDTA (pH 8.0), 20% sucrose), and then the samples were incubated for 60 min at 37 °C. The next steps followed the manufacturer’s instructions. After extraction, samples were stored at −80 °C until the time of use. After total DNA extraction, the samples were quantified using the Qubit Fluorometer equipment (Thermo Fisher Scientific, Waltham, MA, USA).

### 2.4. 16S rRNA Gene Sequence Processing

The characterization of the newborn’s saliva microbiota was performed by amplification of the V3-V4 domain of the bacterial 16S ribosomal segment, which was selected from the work carried out by Klindworth et al. [24]. The full-length primers using standard IUPAC nucleotide nomenclature to follow the protocol for this region are: V3-V4 forward primer (5′-TCG TCG GCA GCG TCA GAT GTG TAT AAG AGA CAG CCT ACG GGN GGC WGC AG-3′) and V3-V4 reverse primer (5′-GTC TCG TGG GCT CGG AGA TGT GTA TAA GAG ACA GGA CTA CHV GGG TAT CTA ATC C-3′). All procedures were performed following the manufacturer’s protocol (Illumina-16S Metagenomic Sequencing Library Preparation). The size of the PCR fragment is 550 bp and 630 bp after indexing the adapters. We used the Nextera XT adapters and V2 500 cycle sequencing reagent kit. Subsequently, the samples were sequenced using the Illumina MiSeq platform (Illumina^®^, San Diego, CA, USA), according to the manufacturer’s instructions.

### 2.5. Data Analysis

After obtaining the sequences, the 16S rRNA libraries were analyzed using the QIIME v.2-2020.2 software [25]. Denoising was performed with DADA2 [26]. The forward sequences were then truncated at position 251, while the reverse sequences were truncated at 250 nucleotides, in order to discard positions for which the median nucleotide quality was less than Q30. Samples with less than 1000 sequences were also excluded from further analyses. The taxonomy was assigned using ASVs (Amplicon Sequencing Variant) through the q2-feature-classifier resource [27] and the Bayes naive classify-sklearn taxonomy classifier, comparing the obtained ASVs against the SILVA reference database 132 [28]. Subsequent analyses were performed with the R version 4.0.4 using the *phyloSeq* [29], *vegan* [30], *microbiome* [31], and *ggplot2* [32] packages.

### 2.6. Statistical Analyses

Statistical analyses were performed using IBM SPSS Statistics for Windows, version 26 (IBM Corp., Armonk, N.Y., USA) and R Studio version 1.4.1106 (R version 4.0.4) (R Foundation for Statistical Computing, Vienna, Austria). The described data analysis was performed using the Generalized Linear Model (GzLM) with linear distribution for comparison between numerical variables and binary logistic distribution for categorical variables (Figure S1). The Alpha diversity was measured by the indices (Chao1, Shannon, Simpson, and Faith’s phylogenetic diversity) and the difference between groups over time were performed by the Generalized Estimating Equations (GEE), in which the models were evaluated as gamma or linear distribution and identity linkage function, adjusting for type of delivery (cesarean or vaginal), gestational age, and antibiotic use (yes or no). Furthermore, the correlation matrix varied between independent, AR (1), unstructured, and exchangeable. In order to choose the best model, the lowest Quasi likelihood under Independence Criterion (QIC) was considered and the best adherence of residues [33] was also evaluated using the Q-Q plot [34] (Figure S2). For differences in beta diversity of the groups over time, PERMANOVA (adonis test; *vegan* R package) was performed using the weighted, unweighted, and generalized UniFrac distances, and also Bray–Curtis dissimilarity to compare the groups (OAC and SC) by time (T0 to T3), using 999 permutations. To compare genera relative abundances between the groups over time, a GAMLSS-BEZI (Generalized Additive Models for Location, Scale, and Shape with a zero inflated beta family) model was used (*metamicrobiomeR* package [35]). The models were adjusted for confounders variables [mode of delivery (cesarean or vaginal), gestational age (weeks), and infant antibiotic use (yes or no)]. To be accounted in the model, genera were filtered for relative abundance higher than 0.0005 and prevalence higher the 0.05. Multiple comparisons were controlled by using a false discovery rate adjustment. A *p* value < 0.05 was considered significant.

### 2.7. Data Deposition

Sequence data have been deposited in the National Center for Biotechnology Information (NCBI) under BioProject ID PRJNA762545.

## 3. Results

Among 108 premature newborns admitted to the NICU during the sample collection period, 24 were included in the study. Of these, two were excluded, one full-term newborn and one newborn who was lost to follow-up. In addition, two premature newborns who died during hospitalization were not included. Thus, 20 newborns entered the analysis stage, 11 in the group receiving oropharyngeal administration of colostrum (OAC group), and nine in the group receiving standard care without the administration of colostrum (SC group). During the first week of life, in addition to standard care or colostrum administration, both groups received a diet, either orally or by orogastric tube. The SC group, despite not receiving the colostrum protocol, received, in low proportions, oral diet during this period. Table S1 shows the percentage of oral stimulation (either oropharyngeal administration of colostrum or oral diet) in the groups. We can observe that the oral stimulus was much higher in the OAC group (73.3%) compared to the SC group (22.3%), and that the use of donor breast milk was higher than the use of mother’s milk in both groups.

A total of 79 samples were collected from this population, and after bioinformatics analysis, five were excluded for having low read count (<1000 reads); thus, 74 were used for statistical analyses.

### 3.1. Descriptive and Clinical Data

Descriptive and clinical data from the groups can be found in Table 1. Regarding the characteristics of the mothers of the participating newborns, we did not observe a difference in the mean maternal age (*p* = 0.27) and in the obstetric history (*p* = 0.21) between the groups. No differences were found for gestational age at birth, sex, mode of delivery, sepsis occurrence, antibiotic therapy, days spent in the NICU, or time on parenteral nutrition comparing the groups. We found differences for birth weight, which was significantly higher in the SC group compared to the OAC group (*p* = 0.013). In addition, the SC group also had a significantly higher weight on the 7th day of life compared to the OAC group (*p* = 0.017). In the other weight comparisons between the groups (14th and 21st day after birth and at hospital discharge), no differences were observed.

### 3.2. Relative Abundance

**Figure 1:**
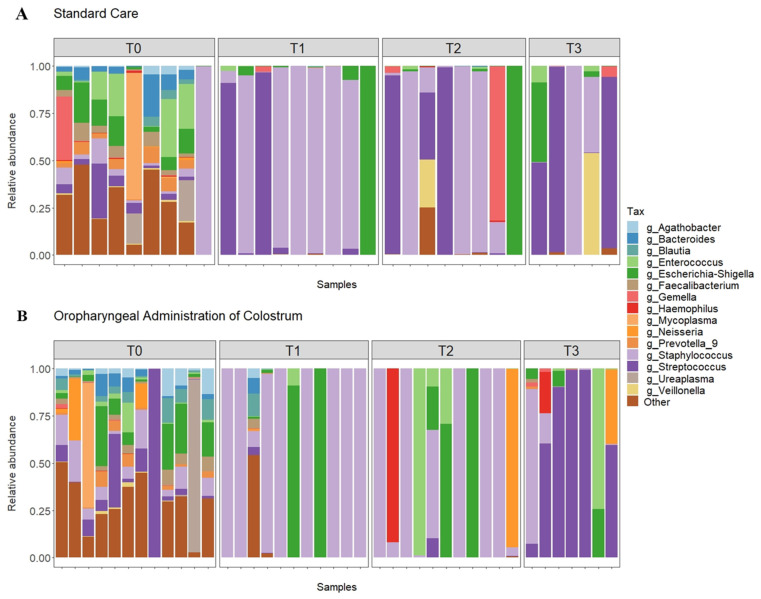
Relative abundance of top 15 genera in all samples from the SC (**A**) and OAC (**B**) groups over time. Legend: OAC (Oropharyngeal Administration of Colostrum); SC (Standard Care); T0 (first day of life), T1 (7 days after birth), Time 2 (14 days after birth), Time 3 (21 days after birth).

**Figure 2:**
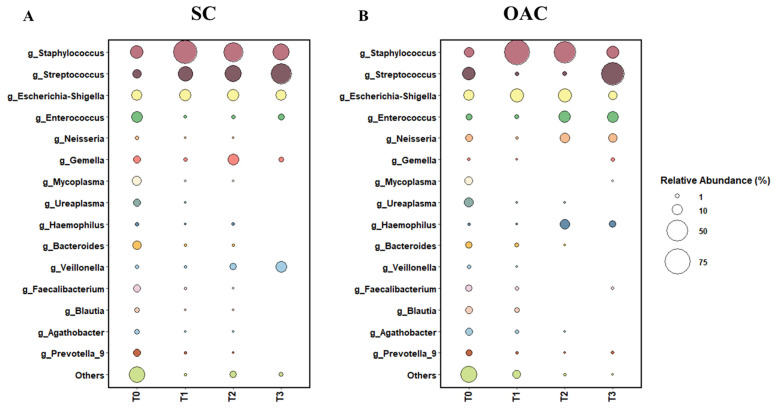
Relative abundance of top 15 genera between SC (**A**) and OAC (**B**) groups over time. Legend: OAC (Oropharyngeal Administration of Colostrum); SC (Standard Care); T0 (first day of life), T1 (7 days after birth), Time 2 (14 days after birth), Time 3 (21 days after birth).

The statistical analyses and distribution of the 15 most abundant bacterial genera in saliva samples in each collection time (1st, 7th, 14th, and 21st day of life) can be seen by sample in Figure 1 (1A-SC group and 1B-OAC group), by groups in Figure 2 (2A-SC group and 2B-OAC group), and the significant results in Table S2.

The most prevalent genera on the first day of life (T0) were *Staphylococcus*, *Enterococcus*, *Escherichia-Shigella*, and *Streptococcus*. When comparing the groups, we observed a greater abundance of *Staphylococcus* and *Enterococcus* in the SC group, while the OAC group had a greater abundance of *Streptococcus* and *Escherichia-Shigella*; however, the results were not statistically significant. In contrast, we observed a significantly greater abundance of *Agathobacter* (*p* = 0.041) and *Blautia* (*p* = 0.013) in the OAC group compared to the SC group, and a trend towards a greater abundance of *Haemophilus* in the SC group compared to the OAC group (*p* = 0.051). Comparing the groups on the 7th day of life (T1), we identified a greater abundance of *Staphylococcus* and *Escherichia-Shigella* in the OAC group and a greater abundance of *Streptococcus* in the SC group, but the results did not reach statistical significance. However, we observed a significantly higher abundance of *Bifidobacterium* in the OAC group compared to the SC group (*p* < 0.001), even though this genus was presented in low abundance in both groups (out of the top 15).

On the 14th day of life (T2), the most dominant genera were *Staphylococcus*, *Escherichia-Shigella*, and *Streptococcus* in the SC group; in the OAC group, *Staphylococcus*, *Escherichia-Shigella*, and *Enterococcus* were the most abundant genera. In the comparison between the groups, we identified a significantly greater abundance of *Haemophilus* in the OAC group compared to the SC group (*p* < 0.001). On the 21st day of life (T3), the most dominant genera in both groups were the same as observed in T2. In the comparison between the groups, we identified a significantly greater abundance of *Gemella* in the SC group compared to the OAC group (*p* = 0.001).

### 3.3. Comparison between Groups after Administration of Colostrum

**Figure 3:**
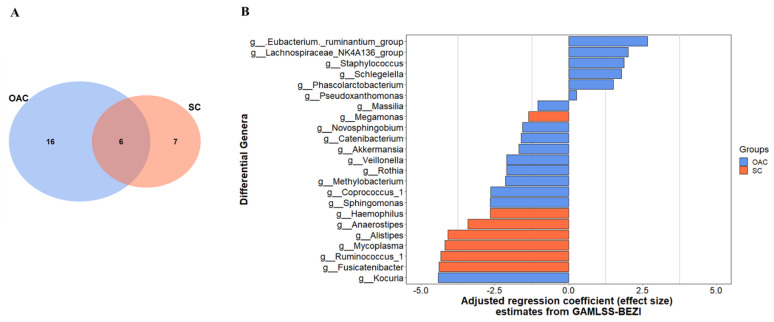
Differential genera between groups in the 7th day of life (T1) comparing to the first day of life (T0); shared and unique genera between groups (**A**), and the adjusted regression coefficient estimates from GAMLSS-BEZI (**B**). Legend: GAMLSS-BEZI (Generalized Additive Models for Location, Scale, and Shape with a zero inflated beta family); OAC (Oropharyngeal Administration of Colostrum); SC (Standard Care).

When comparing the groups after colostrum administration from the first day of life to the 7th day after delivery (Figure 3), we observed that changes occurred in both groups. Six genera, including *Faecalibacterium*, *Lactobacillus*, and *Prevotella 9* decreased in both groups. We identified major changes in OAC than SC, since 16 genera were different from T0 to T1, compared to seven in SC (Figure 3A). Among the genera that changed in OAC, 10 decreased in T1, such as *Sphyngomonas*, *Veillonella*, and *Rothia*, while six genera increased in the same period, including *Eubacterium*, *Lachnospiraceae*, and *Staphylococcus*. In the SC group the *Ruminococcus 1*, *Mycoplasma*, and *Alistipes* are among the genera that decreased in T1 whereas no genera had increased (Figure 3B).

### 3.4. Alpha Diversity

**Figure 4:**
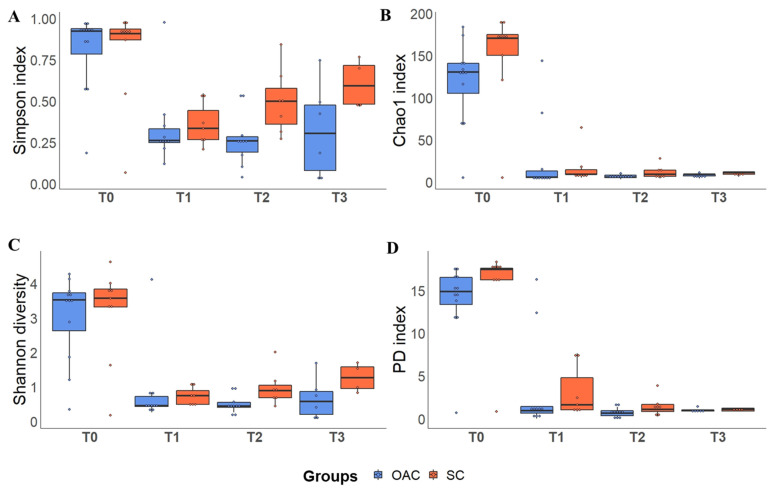
Alpha diversity indices (Simpson—(**A**); Chao 1—(**B**); Shannon—(**C**) and PD—(**D**)) between groups over time. Boxplots show the median, 25% percentile and 75% percentile of the diversity indexes of each group. Dots represent each sample. Legend: PD: Faith’s Phylogenetic Diversity Index; T0 (first day of life), T1 (7 days after birth), Time 2 (14 days after birth), Time 3 (21 days after birth).

The results of the alpha diversity indices in the groups in relation to time can be seen in Figure 4 and in Table S3. Regarding the Chao1 richness index, we observed a significant decrease in the comparison between T0 and all the other analysis time points (*p* = 0.001). In the interaction between groups and time, it is possible to identify a trend towards greater Chao1 richness (*p* = 0.078). In the comparison between groups and time for the Shannon diversity index, we observed a decrease between T0 and all the other analyzed time points (*p* < 0.001). No difference was observed in the analysis between groups at T0, but it was possible to identify a significant decrease in this index between T0 and T1 in both the OAC and SC groups (*p* < 0.01). In the SC group, a significant decrease was also observed when comparing the value found at T1 with that found at T3 (*p* < 0.001). Furthermore, there was a significant difference between the groups at T3, with higher values for the Shannon index in the SC group compared to the OAC group (*p* = 0.015).

In the Simpson’s index analysis, we also identified a significant decrease at T0 in relation to the other analysis time points (*p* < 0.001). Furthermore, there was a significant increase in T3 compared to T2, regardless of the group. In the interaction between group and time, no differences were observed between the groups at baseline, and both groups showed a significant decrease from T0 to T1 (*p* < 0.001) and from T0 to T2 (*p* < 0.001 in the OAC group and *p* = 0.029 in the SC group). In the OAC group, we observed a decrease at T3 compared to T0 (*p* = 0.015). On the other hand, the SC group showed a significant increase in this index at T3 compared to T1 (*p* < 0.001). In the evaluation of the Faith’s phylogenetic diversity index, a decrease in diversity was observed in both groups over time (*p* < 0.001). In the interaction between group and time, there were no differences between the groups at T0, and both groups showed a significant decrease from T0 to T1, T2, and T3 (*p* < 0.001).

### 3.5. Beta Diversity between Groups and after the Intervention

**Figure 5:**
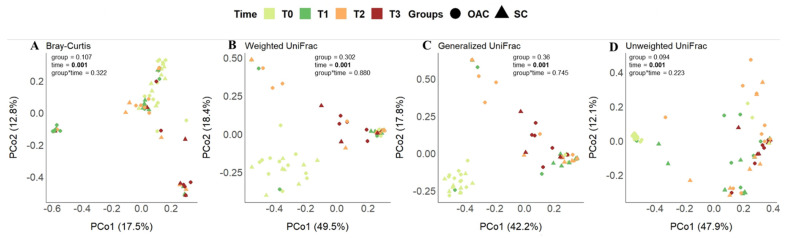
Beta diversity distances (Bray–Curtis—(**A**); Weighted UniFrac—(**B**); Generalized UniFrac—(**C**) and Unweighted UniFrac—(**D**)) between groups over time. Legend: OAC (Oropharyngeal Administration of Colostrum); SC (Standard Care); T0 (first day of life), T1 (7 days after birth), Time 2 (14 days after birth), Time 3 (21 days after birth).

In the principal coordinates analysis (PCoA) considering the difference between groups (Figure 5), we found no differences in the phylogenetic structure between the groups over time, in any of the metrics analyzed. The same was observed for the interaction between group and time; however, when only the effect of time on the samples was considered, we found significant results for all metrics (*p* = 0.001). Thus, it is possible to identify, in this sample and population, how time changes the dynamics of the structure of this community.

**Figure 6:**
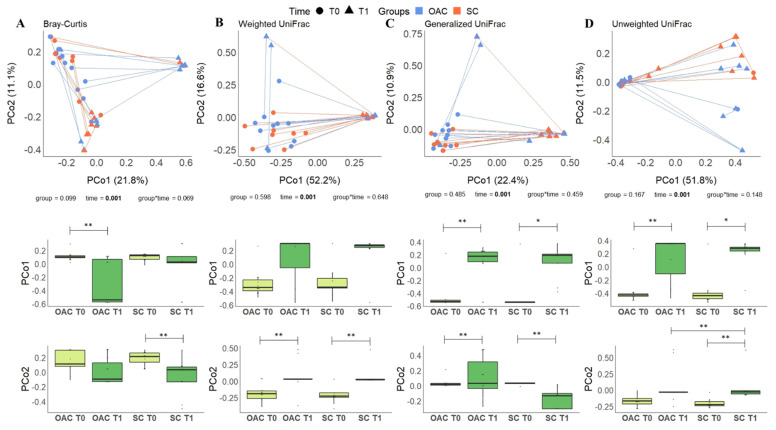
Beta diversity distances (Bray–Curtis—(**A**); Weighted UniFrac—(**B**); Generalized UniFrac—(**C**) and Unweighted UniFrac—(**D**)) comparing OAC group (blue) to SC group (red) between baseline (T0—circles) and after the protocol (T1—triangles); * Indicates a *p* value between 0.01 and 0.05; ** Indicates a *p* value between 0.01 and 0.001. Legend: OAC (Oropharyngeal Administration of Colostrum); SC (Standard Care).

Regarding the analysis after the intervention (T1) compared to the day of birth (T0), the results can be seen in Figure 6. Similar to previous analysis, no differences were found in the comparison between groups with the metrics used. In the analysis of the interaction between group and time, it was possible to observe a trend towards significance in the Bray–Curtis index (*p* = 0.069); in the analysis considering only the effect of time, once again we found significant results for all metrics (*p* = 0.001).

## 4. Discussion

The beginning of life and the factors involved in this phase are extremely relevant for the development of newborns, whether premature or full term. Nowadays, it is known that the pregnant woman’s weight gain, diet, and type of delivery directly influence the newborn’s health. In this sense, breastfeeding is part of the development of the immune system and colonization of the gastrointestinal tract of newborns, through bacteria present in breast milk. In this context, the importance of breast milk for the health of newborns has already been well documented [36]. However, premature neonates are often unable to be breastfed, as they need to be hospitalized in intensive care units and oral feeding is limited.

Considering the importance of breast milk for the nutrition of preterm newborns, practices such as the oropharyngeal administration of colostrum help in the development of immunity and in the oral colonization of this population. Recent studies have shown that this practice is safe [37], reduces the length of hospital stay [38], and the risk for the development of necrotizing enterocolitis [39]. It has already been reported that prebiotics stimulate the growth of beneficial bacteria in the oral cavity, helping to maintain microbial balance [40]. In addition, breastfeeding influences the transfer of specific bacteria from breast milk to the baby’s intestine, modifying the composition of the intestinal microbiota and helping its development [41,42].

In the saliva samples collected from preterm newborns, we observed that on the first day of life, the groups presented greater diversity of bacterial genera, with no great predominance of any specific genus, as can be seen in the results of the alpha diversity indices. After the protocol (T1–7th day), we observed an increase in *Staphylococcus* in both groups compared to T0; however, this increase was greater in the OAC group compared to the SC group, probably due to greater oral stimulation, either by colostrum administration or by oral diet. This result supports the concept of bacteria transfer from breast milk to the newborn, as the most abundant genus observed in breast milk samples was *Staphylococcus*. In this context, the oral microbiota plays an important role in the colonization of the intestinal microbiota, as it is an obligatory passage for breast milk to reach the gastrointestinal tract [43]. The oral microbiota of healthy and full-term newborns is generally dominated by *Streptococcus* and *Staphyloccocus*, and may have, in smaller proportions, genera such as *Gemella*, *Actinomyces*, and *Veillonella* [44]. The oral cavity is constantly exposed to the environment, changing continuously, along with the constant production of saliva that creates a continuous flow. Thus, the formation of biofilm on the oral surface facilitates the colonization and formation of the oral microbiota [45].

In addition to *Staphylococcus*, one of the most abundant genera found in the samples from this newborn population was *Streptococcus*, from the first day of life, but mainly to the 21st day of life, when it accounts for half of the relative abundance observed in both groups, becoming the most prevalent genus in this environment. *Streptococcus* present in the oral mucosa are known as one of the main groups of early colonizers and their adhesion to the oral cavity promotes the establishment of later colonizers, through their production of polysaccharides and adhesins [45]. Evidence shows that there is a similarity in the composition of the oral microbiota and the intestinal microbiota of newborns, which suggests that bacteria that cannot adhere to the oral surface and are swallowed participate in intestinal colonization [46,47].

Specifically for preterm infants, few studies have evaluated the development of oral microbiota in this population, mostly through culture methods. A study carried out by Hendricks-Muñoz et al. (2015) with premature newborns observed that the oral microbiota after 1 month of life was dominated by the genus *Streptococcus*, associating the presence of other bacterial genera with skin-to-skin contact after birth [48]; however, the study did not analyze the effect of breastfeeding or the administration of colostrum.

A study by Sohn et al. (2015) evaluated the effect of oral administration of colostrum on the oral microbiota of extremely low birth weight newborns. They found a difference in the oral microbiota from birth to the 4th day of life, with a prevalence of the Moraxellaceae family at birth and the Planococcaceae family after the protocol. In the group that did not receive colostrum, they observed a greater abundance of the Staphylococcaceae family on the 4th day of life [49]. However, this study did not assess the microbiome composition of the colostrum administered, and considered the results at the family phylogenetic level only. Contrasting results were found in our study, in which the most abundant genus in both groups was *Staphylococcus*, which belongs to the Staphylococcaceae family. The differences found may be due to the methodology used or due to some specific characteristic of the population. In the study by Ruiz et al. (2019), in addition to the pre-colostrum composition, the oral microbiota of newborns 5 to 7 days after birth was observed. *Staphylococcus* and *Streptococcus* were the most abundant genera found in the oral microbiota of newborns [50], which is in line with the findings of the present work.

On the 7th day of life, in the group receiving colostrum, there was an increase in the genera *Bifidobacterium* and *Bacteroides*, both known as initial colonizers of the intestinal microbiome. *Bifidobacterium* species are responsible for metabolizing oligosaccharides [51], important components for healthy colonization in the intestinal environment, and *Bacteroides* are responsible for breaking down complex molecules in the intestine, helping the immune system against any pathogens [52]. In our sample, these genera were found to transiently colonize the oral environment. This may be because, in our population, neonates in the OAC group received a large amount of oral stimulation in the first week of life, while the control (SC) group had a low proportion of oral stimulation during the entire hospitalization period.

In the post-protocol period, we observed an increase in *Neisseria* and *Haemophilus* in the OAC group. In the SC group, we identified a significant increase in *Gemella* at T3 (21st day of life). *Neisseria*, *Haemophilus*, and *Gemella* are bacterial genera commonly found in the oral microbiome, either in newborns or in adults. However, these genera were not reported in the work carried out by Young et al. (2020) with seven extremely premature newborns (mean gestational age of 23.6 weeks), which identified a greater abundance of *Staphylococcus* and *Escherichia-Shigella* in the 3rd–4th week of life [53].

Romano-Keeler et al. (2017) carried out a study with preterm newborns (<32 complete weeks of gestation) to observe the effects of a priming with oral colostrum. The protocol consisted of administering 100 µL of colostrum from the mother in each cheek every 6 h for 5 days, starting in the first 48 h of life [38]. In their results, they found no differences between groups in the Shannon index, but the diversity decreased over time, between the 1st and the 30th day of life. Additionally, they found no differences in beta diversity in relation to treatment, but observed that the community profile changed over time [38]. Similar results were observed in our study, in which the alpha diversity indices decreased between the 1st and the 7th day of life, which lasted until the 21st day of life. Furthermore, no differences were observed between groups in the beta diversity analysis, but it was possible to verify the effect of time on the phylogenetic profile of oral samples.

The present study had some limitations. The small sample size made it difficult to extrapolate the findings. In addition, because it is an observational study and composed of a convenience sample, it was not possible to present a group receiving only the protocol with the mother’s milk, since it is extremely difficult to control or predict the food that will be offered to premature newborns in a NICU. The loss of follow-up of some samples is also noteworthy, as it impacts the statistical power of the study.

## 5. Conclusions

This study demonstrated that protocols using the oropharyngeal administration of colostrum are important for the colonization of the oral microbiota, as they promote an early maturation of the oral cavity, which can be observed by the bacterial profile found after colostrum administration, with increased abundance of beneficial bacteria, such as *Staphylococcus*, *Bifidobacterium*, and *Bacteroides*. In addition to the effects of this protocol, we observed that the newborn’s oral microbiota is dynamic, and changes over time. Therefore, extensive trials are needed and essential to promote the inclusion of this intervention as a standard protocol for preterm infants.

## Figures and Tables

**Table 1:** Comparison of descriptive and clinical data between groups (*n* = 20).

Variable	OAC Group (*n* = 11)	SC Group (*n* = 9)	Wald Chi-Square	*p* Value
Maternal age (years) ^#^	29.9 ± 4.8	27.1 ± 7.3	1.178	0.27
Gestational age (weeks) ^#^	30.6 ± 2.3	31.4 ± 1.7	0.736	0.39
Obstetric history ^§^			1.546	0.21
Multiparous	9 (81.8)	5 (55.6)		
Primiparous	2 (18.2)	4 (44.4)		
Sex ^§^			0.884	0.34
Female	5 (45.5)	6 (66.7)		
Male	6 (54.5)	3 (33.3)		
Type of delivery ^§^			0.020	0.88
C-section	7 (63.6)	6 (66.7)		
Vaginal	4 (36.4)	3 (33.3)		
Sepsis ^§^			1.135	0.28
Yes	5 (45.5)	2 (22.2)		
No	6 (54.5)	7 (77.8)		
Use of antibiotics on NICU ^£^			0.050	0.82
Yes	9 (81.8)/16.2 (2–30)	7 (77.8)/9.8 (2–27)		
No	2 (18.2)	2 (22.2)		
Parenteral nutrition time (days)	16.1 ± 10.8	10.7 ± 11.4	1.309	0.25
Birth weight (grams)	1180.8 ± 249.2	1493.3 ± 344.4	6.159	0.013 *
Weight on the 7th day of life (grams)	1140.1 ± 250.5	1416.0 ± 297.2	5.649	0.017 *
Weight on the 14th day of life (grams)	1278.8 ± 319.5	1529.5 ± 281.6	3.760	0.052
Weight on the 21st day of life (grams) ^¥^	1372.2 ± 298.3	1570.1 ± 307.8	2.069	0.15
Weight at hospital discharge (grams)	1963.0 ± 237.8	2055.0 ± 153.9	1.067	0.30
Days in the NICU	44.1 ± 32.2	23.4 ± 23.2	2.868	0.09

Values are expressed as ^#^ mean ± standard deviation; ^§^ number (percentage); ^£^ number (percentage)/mean (minimum-maximum). *p* values are based on Generalized Linear Model (GzLM) with ^#^ linear distribution and ^§, £^ binomial distribution. * Significance ≤ 0.05. Legend: OAC (Oropharyngeal Administration of Colostrum); SC (Standard Care); NICU (Neonatal Intensive Care Unit). ^¥^ Two newborns were discharged from the hospital before completing 21 days of hospitalization.

## Data Availability

Sequence data have been deposited in the National Center for Biotechnology Information (NCBI) under BioProject ID PRJNA762545.

## Supplementary Materials

The following are available online at https://www.mdpi.com/article/10.3390/nu13124224/s1, Figure S1: Q-Q plot in relation to the adherence residues of the descriptive data between groups, Figure S2: Q-Q plot in relation to the adherence residues of the alpha diversity indices: Chao 1 (A), Shannon (B), Simpson (C), and Faith’s Phylogenetic Diversity (D) for the analysis of the oral microbiota, Table S1: Percentage of oral stimulus (either diet or oropharyngeal administration of colostrum) during the first week of life, Table S2: Comparison of the main genera between groups over time, Table S3. Alpha diversity indices considering the interaction between group/time.
