# Supplementary material for: Impact of Oropharyngeal Administration of Colostrum in Preterm Newborns’ Oral Microbiome
Source: Nutrients. 2021 Nov 24;13(12):4224. doi: 10.3390/nu13124224 (PMC8703686; doi:10.3390/nu13124224)
Supplement: Supplementary file 1 [file nutrients-13-04224-s001.zip › nutrients-1398483-supplementary.pdf]

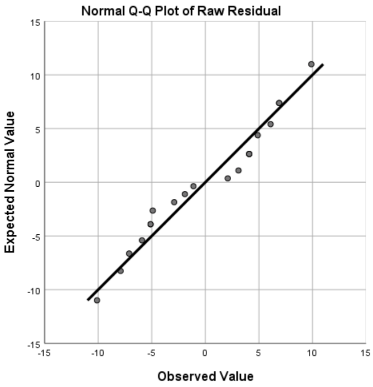


(**A**) Maternal age

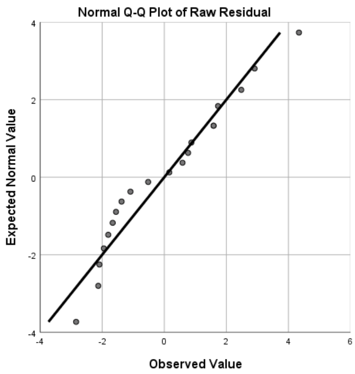


(**B**) Gestational age

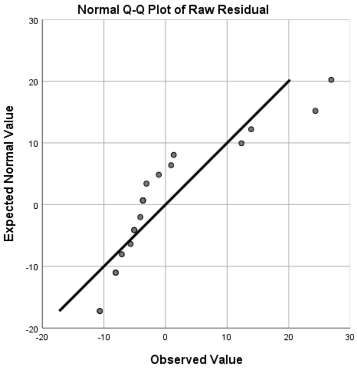


(**C**) Parenteral nutrition time

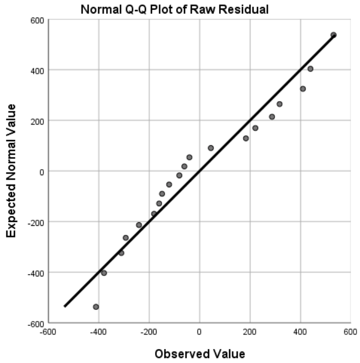


(**D**) Birth weight

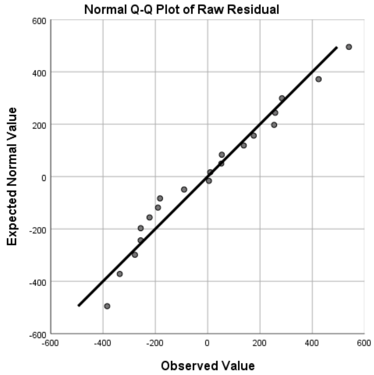


(**E**) Weight on the 7th day of life

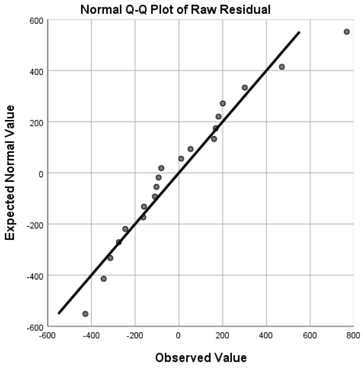


(**F**) Weight on the 14th day of life

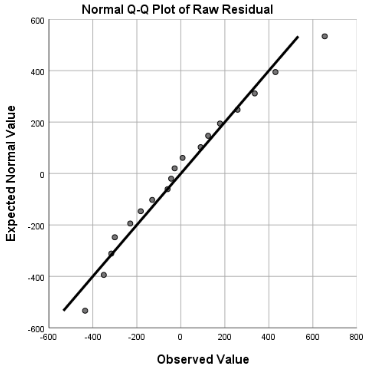


(**G**) Weight on the 21th day of life

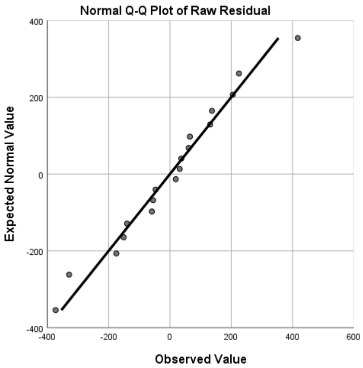


(**H**) Weight at hospital discharge

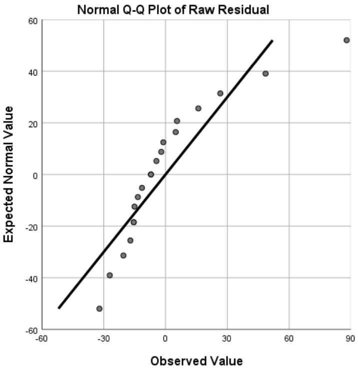


(**I**) Days in the NICU

**Figure S1.** Q-Q plot in relation to the adherence residues of the descriptive data between groups. **Legend:** Maternal age (**A**), Gestational age (**B**), Parenteral nutrition time (**C**), Birth weight (**D**), Weight on the 7th day of life (**E**), Weight on the 14th day of life (**F**), Weight on the 21th day of life (**G**), Weight at hospital discharge (**H**), and Days in the NICU (I). NICU (Intensive Care Unit).

For all analyses, the linear distribution was chosen with identity link function.

(**A**) Chao1

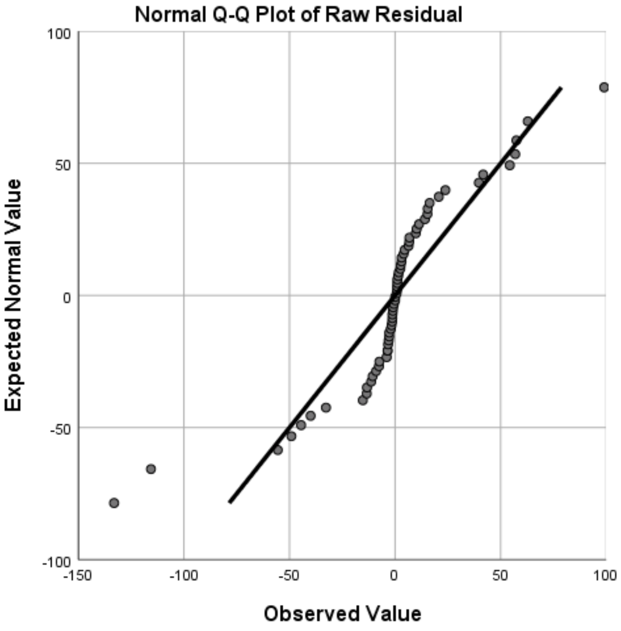


(**B**) Shannon

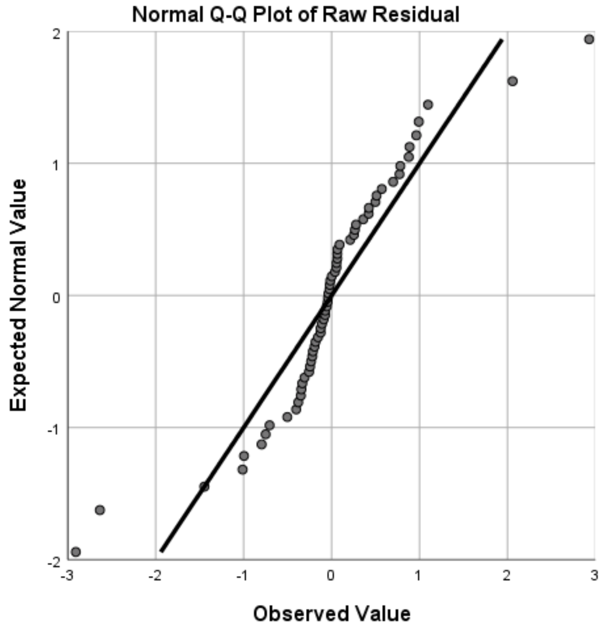


Distribution: Gamma
Link function: Identity
Covariance matrix: Independent

Distribution: Linear
Link function: Identity
Covariance matrix: AR(1)

(**C**) Simpson

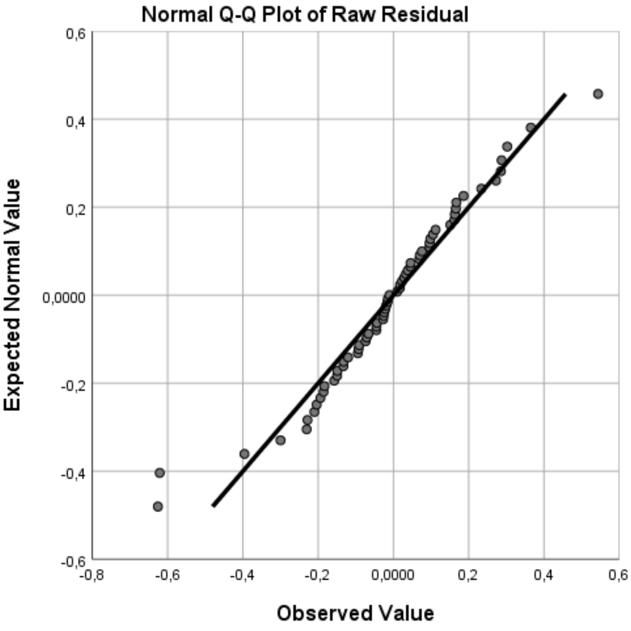


(**D**) Faith's Phylogenetic Diversity

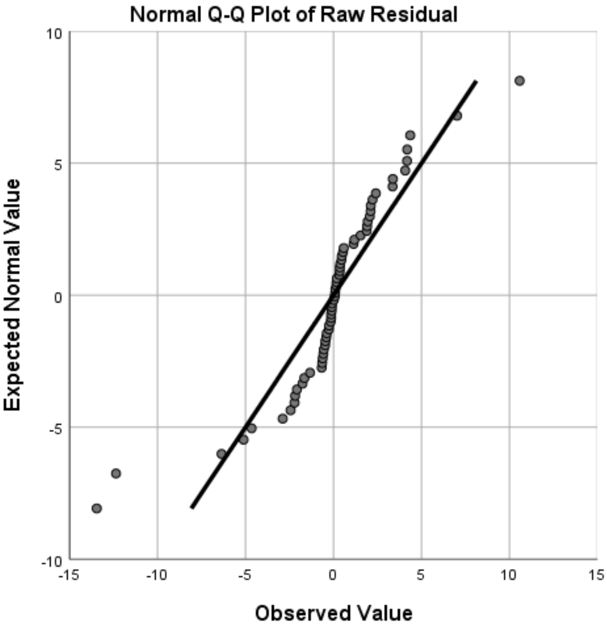


Distribution: Linear
Link function: Identity
Covariance matrix: Unstructured

Distribution: Gamma
Link function: Identity
Covariance matrix: Exchangeable

**Figure S2.** Q-Q plot in relation to the adherence residues of the alpha diversity indices. **Legend:** Chao 1 (**A**), Shannon (**B**), Simpson (**C**) and Faith's Phylogenetic Diversity (**D**) for the analysis of the oral microbiota.

**Table S1.** Percentage of oral stimulus (either diet or oropharyngeal administration of colostrum) during the first week of life.

| Type of Breast Milk | OAC Group (n = 11) | SC Group (n = 9) |
|---|---|---|
| Mother's own milk | 15.9 ± 9.4 | 2.9 ± 5.5 |
| Donor breast milk (pasteurized) | 57.4 ± 15.3 | 19.4 ± 16.7 |
| Mean percentage | 73.3% | 22.3% |

Values presented as mean percentage ± standard deviation. Infants did not receive infant formula during the first week of life.

**Table S2.** Comparison of the main genera between groups over time (n = 20).

| T0 | OAC Group (n = 11) | SC Group (n = 9) | Estimate | *P* Value | FDR Adjusted P Value |
|---|---|---|---|---|---|
| *Agathobacter* | 1212.44 ± 478.02 | 574.03 ± 206.08 | −1.101 | <0.01 | 0.019 * |
| *Blautia* | 1096.62 ± 327.07 | 650.99 ± 243.15 | −1.364 | <0.01 | 0.04 * |
| **T1** | | | | | |
| *Bacteroides* | 362.36 ± 313.72 | 24.79 ± 19.88 | −7.843 | 0.02 | 0.06 |
| *Bifidobacterium* | 1654.31 ± 1465.43 | 23.45 ± 19.62 | −19.508 | <0.001 | <0.001 * |
| **T2** | | | | | |
| *Bacteroides* | 1.89 ± 1.50 | 7.47 ± 5.65 | 3.024 | <0.01 | 0.12 |
| *Haemophilus* | 2752.87 ± 2443.06 | 102.67 ± 57.56 | −7.078 | <0.001 | <0.001 * |
| **T3** | | | | | |
| *Gemella* | 70.40 ± 55.62 | 791.04 ± 564.86 | 0.014 | <0.001 | <0.001 * |

Values are expressed as mean ± standard error. * Significance ≤ 0.05. P values were based on GAMLSS-BEZI (Generalized Additive Models for Location, Scale and Shape with a zero inflated beta family). Values adjusted by type of delivery (c-section or vaginal), gestational age, and use of antibiotics (yes or no). **Legend:** OAC (Oropharyngeal Administration of Colostrum); SC (Standard Care); FDR (False Discover Rate); T0 (first day of life), T1 (7 days after birth), Time 2 (14 days after birth), Time 3 (21 days after birth).

**Table S3.** Alpha diversity indices considering the interaction between group/time

| Indices | Mean ± Standard Error | | Tests of Model Effects | | | | | |
|---|---|---|---|---|---|---|---|---|
| | | | Time | | | Interaction | | |
| | OAC Group (n = 11) | SC Group (n = 9) | Wald Chi-Square | DF | P | Wald Chi-Square | DF | P |
| Chao1 # | | | 63.577 | 3 | <0.0001 | 14.165 | 8 | 0.078 |
| Time 0 | 118.92 ± 15.62 | 147.31 ± 19.24 | | | | | | |
| Time 1 | 28.59 ± 13.07 | 16.96 ± 6.09 | | | | | | |
| Time 2 | 6.08 ± 0.71 | 11.91 ± 3.15 | | | | | | |
| Time 3 | 7.99 ± 1.03 | 10.04 ± 1.05 | | | | | | |
| Shannon # | | | 27.703 | 3 | <0.0001 | 47.600 | 8 | 0.0001 * |
| Time 0 | 3.01 ± 0.37 | 3.09 ± 0.46 | | | | | | |
| Time 1 | 0.90 ± 0.34 | 0.75 ± 0.09 | | | | | | |
| Time 2 | 0.48 ± 0.07 | 0.93 ± 0.14 | | | | | | |
| Time 3 | 0.62 ± 0.18 | 1.29 ± 0.07 | | | | | | |
| Simpson § | | | 26.961 | 3 | <0.0001 | 40.257 | 8 | 0.0001 * |
| Time 0 | 0.79 ± 0.07 | 0.77 ± 0.09 | | | | | | |
| Time 1 | 0.37 ± 0.06 | 0.35 ± 0.04 | | | | | | |
| Time 2 | 0.25 ± 0.04 | 0.49 ± 0.06 | | | | | | |
| Time 3 | 0.37 ± 0.06 | 0.66 ± 0.02 | | | | | | |
| Faith's PDI | | | 73.722 | 3 | <0.0001 | 15.976 | 8 | 0.043 * |
| Time 0 | 13.63 ± 1.46 | 15.20 ± 1.81 | | | | | | |
| Time 1 | 3.62 ± 1.60 | 3.20 ± 1.07 | | | | | | |
| Time 2 | 0.73 ± 0.16 | 1.51 ± 0.41 | | | | | | |
| Time 3 | 0.81 ± 0.06 | 0.97 ± 0.15 | | | | | | |

**Legend:** OAC (Oropharyngeal Administration of Colostrum); SC (Standard Care); DF (Degree of Freedom); Faith's PDI (Faith's Phylogenetic Diversity Index); Time 0 (first fecal sample), Time 1 (around 7 days after birth), Time 2 (14 days after birth), Time 3 (last fecal sample). * The significance was confirmed with Sidak's post hoc. All indexes were adjusted by type of delivery (c-section or vaginal), gestational age, and use of antibiotics (yes or no). P-value based on Generalized Estimating Equations (GEE) with # gamma distribution and § linear distribution. In both models, the identity link function was chosen. In the Chao1 index, the independent correlation matrix was chosen; in the Shannon index, AR(1); for Simpson's index, unstructured; and in the Faith's PDI, the exchangeable correlation matrix was chosen. In all models were chosen the minor value of Quasi likelihood under Independence Criterion (QIC) and the best adhesion of residues according to Q-Q plot.
